# The complete chloroplast genome of *Polygala fallax* hemsl. (polygalaceae), a medicinal plant in China

**DOI:** 10.1080/23802359.2026.2677954

**Published:** 2026-06-01

**Authors:** Zongkao Huang, Jie Luo

**Affiliations:** School of Architecture and Environmental Engineering, Shaoxing Institute of Technology, Shaoxing, Zhejiang, China

**Keywords:** *Polygala fallax*, complete chloroplast genome, phylogenetic analysis

## Abstract

*Polygala fallax* Hemsl. is a medicinally important shrub endemic to southern China. In this study, we report the complete sequencing, assembly, and comprehensive characterization of its chloroplast genome using next-generation sequencing technology. The assembled chloroplast genome spans 164,687 base pairs (bp) with an overall GC content of 36.9%, exhibiting the canonical quadripartite structure typical of angiosperms. This architecture comprises a large single-copy (LSC) region of 83,352 bp, a small single-copy (SSC) region of 8,283 bp, and a pair of inverted repeat (IR) regions, each 36,526 bp in length. Annotation revealed a total of 135 genes, including 89 protein-coding genes, 8 ribosomal RNA (rRNA) genes, and 38 transfer RNA (tRNA) genes. Comparative analyses across multiple *Polygala* chloroplast genomes identified seven highly variable regions—*atpH*-*atpI*, *rps4*-*accD*, *ycf15*-*ycf1*, *ndhF*-*rpl32*, *rpl32*-*ccsA*, *ndhE*-*ndhG*-*ndhI*, and *ycf1*-*trnN*—which represent promising molecular markers for species discrimination within the genus. Phylogenetic reconstruction based on chloroplast genome sequences revealed a close evolutionary relationship between *P. fallax* and *P. arillata*, supporting their placement within the same subgenus (*Chamaebuxus*). This study provides valuable genomic resources for future phylogenetic, taxonomic, and conservation studies of *Polygala* species.

## Introduction

*Polygala* L. is the most representative genus of the Polygalaceae family and comprises approximately 50% of the species of the family (Pastore et al. 2019). The genus exhibits remarkable morphological variation, comprising trees, shrubs, and herbaceous forms, with over 600 recognized species (The Plant List 2013; Sun et al. 2026). Numerous *Polygala* species have long been valued in traditional medicine systems across different cultures. *Polygala fallax* Hemsl. (1886) is a shrub endemic to the subtropical regions of south–central and southeastern China (Yang et al. 2016; Wu et al. 2023). Its rhizomes, rich in terpenoid saponins and oligosaccharide esters, have been extensively employed in traditional Chinese medicine for the treatment of various conditions, including cancer, dementia, neurasthenia, and inflammatory disorders (Ma et al. 2003; Chao et al. 2020). Chloroplast genomes are generally characterized by structural stability and high sequence conservation, making them valuable resources for phylogenetic and evolutionary studies. Despite the medicinal importance of *P. fallax*, genomic resources for this species remain limited. Although Zhang et al. (2020) previously utilized the chloroplast genome of *P. fallax* as an outgroup in their investigation of evolutionary relationships within the Leguminosae family, comprehensive analyses of its chloroplast genome architecture and its systematic position within the genus *Polygala* have not yet been conducted. Here, we report the complete chloroplast genome sequence of *P. fallax*, providing essential data for taxonomic clarification, evolutionary studies, and conservation of this medicinally significant species.

## Materials and methods

**Figure 1.**
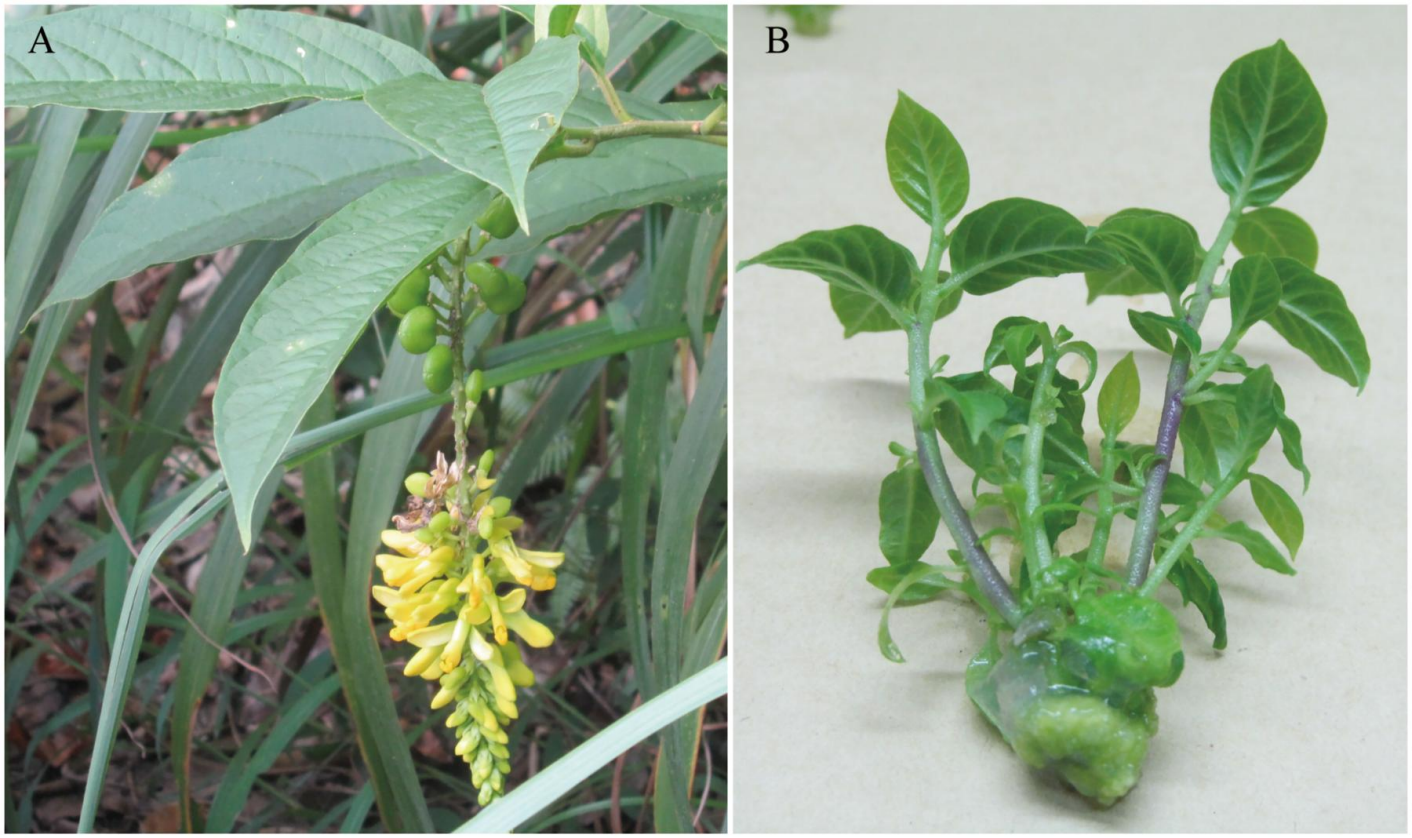
Branch of *P. fallax.* (A) a branch showing the morphology of leaves, flowers, and fruits was collected from Nankun Mountain (113.9812° E, 23.6352° N), identified by: Jie Luo. (B) *In vitro* seedlings of *P. fallax* were obtained *via* tissue culture using seeds as an explant. Jie Luo photographed two pictures above.

Branches of *P. fallax* bearing leaves and seeds (Figure 1) were collected from Nankun Mountain, Guangdong, China (113.9812°E, 23.6352°N). A voucher specimen (SXU-20190415AM03) was deposited at the herbarium of Shaoxing Institute of Technology (Jie Luo, lj26roger@163.com). *In vitro* seedlings were obtained *via* tissue culture using seeds as explants, and total genomic DNA was extracted with the Plant DNA Mini Kit (Genepioneer, Nanjing, China). Short-insert (350 bp) libraries were prepared from the fragmented total genomic DNA following the manufacturer’s protocol and sequenced on the BGISEQ500 platform (BGI, China). A total of 1.8 Gb of raw data (150 bp paired-end reads) was generated. Raw reads were filtered using Trimmomatic (v0.39) to remove low-quality bases (*Q* < 20) to obtain clean data. Cleaned reads were assembled using SPAdes Assembler 3.10.0 (Bankevich et al. 2012). The chloroplast genome was annotated *via* GeSeq (Tillich et al. 2017) and BLAST, and visualized using CPGView (http://www.1kmpg.cn/cpgview, Liu et al. 2023). Multiple sequence alignment was performed with MAFFT v7.450 (Rozewicki et al. 2019). Comparative analyses among five *Polygala* chloroplast genomes (*P. falla*x, *P. hongkongensis*, *P. arillata*, P. *tenuifolia*, *P. japonica*) were conducted using DnaSP v6 (Rozas et al. 2017) and mVISTA (Razer et al. 2004), with nucleotide diversity calculated in 100-bp sliding windows (25-bp step). Phylogenetic analysis was performed using RAxML v8.2.12 (Stamatakis 2014) with the GTR+G + I substitution model, which was selected as the best-fit model *via* jModelTest v2.1.7. To ensure data matrix completeness, 49 common single-copy protein-coding genes present across all 32 species were concatenated for tree reconstruction. The complete chloroplast genome sequence of *P. fallax* was submitted to GenBank with accession number MT762166.

## Results

**Figure 2.**
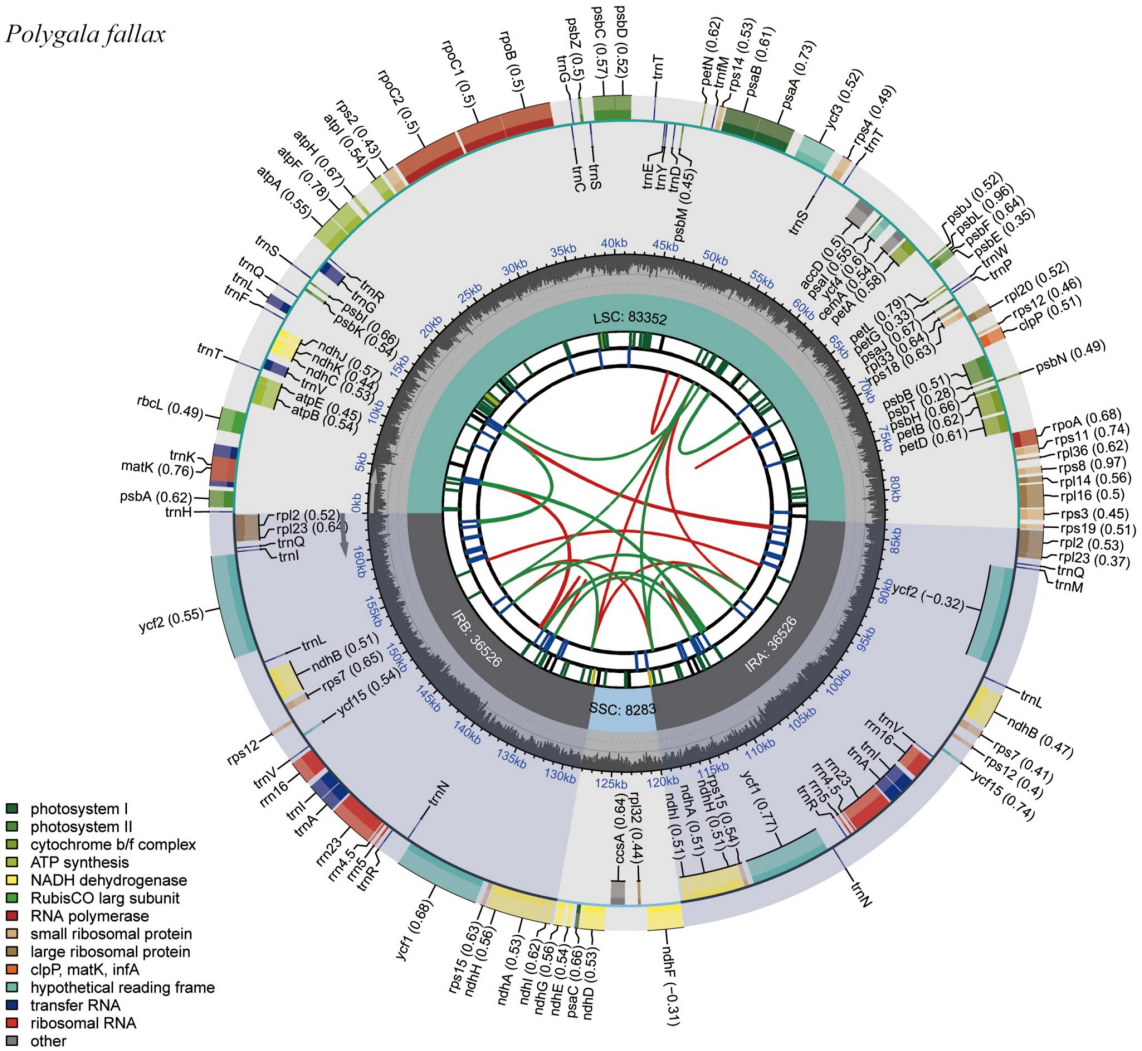
Chloroplast gene map of *P. fallax*. The plastome is displayed in six concentric circles. From the inside out: (1) dispersed repeats (red: forward; green: reverse); (2) tandem repeats (blue bars); (3) simple sequence repeats (SSRs; green bars); (4) boundaries of LSC, SSC, and IR regions; (5) GC content (dark gray = GC, light gray = background); (6) annotated genes, color-coded by functional group. Genes on the outer side of the circle are transcribed clockwise; those on the inner side, counter-clockwise.

The complete chloroplast genome of *P. fallax* was 164,687 bp in length and had a GC content of 36.9%. The complete chloroplast genome showed a typical quadripartite structure that included a large single-copy (LSC) region (83,352 bp), a small single-copy (SSC) region (8,283 bp), and a pair of inverted-repeat (36,526 bp each) regions. The average and minimum read mapping depth of the assembled plastome were 139× and 47×, respectively (Figure S1). 135 genes were identified from the *P. fallax* chloroplast genome, including 89 protein-coding, 8 rRNA, and 38 tRNA genes. Notably, 11 protein-coding, 7 tRNA, and 4 rRNA genes were duplicated in the IR regions. Twenty-two genes contained two introns, and one gene (*ycf3*) contained three introns (Figure 2). Figure S2 and S3 illustrate the complex gene structures challenging annotation in the *P. fallax* chloroplast genome, including cis-splicing genes with exon-intron organization and the trans-splicing rps12 gene split across LSC and IR regions.

**Figure 3.**
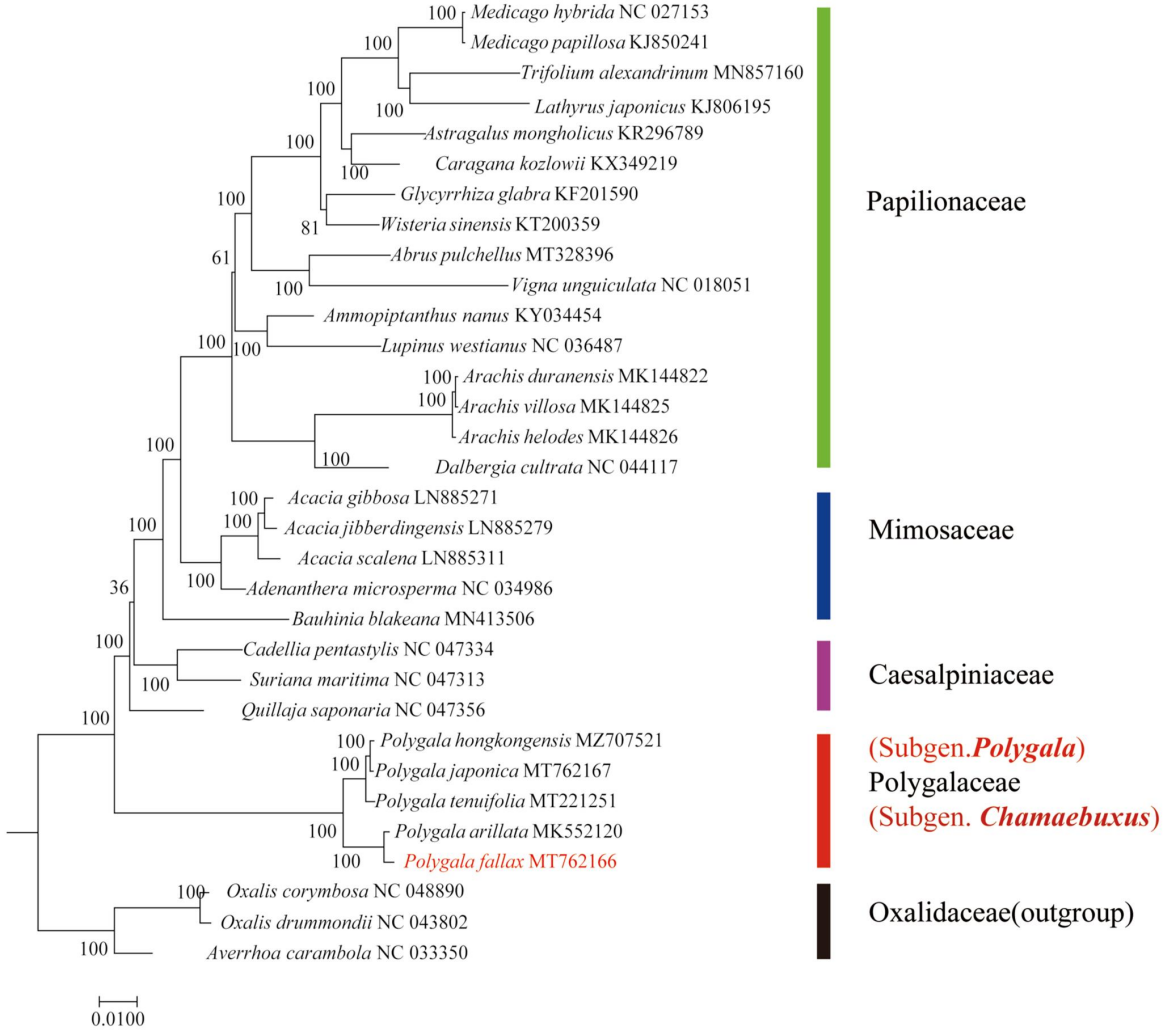
Maximum-likelihood (ML) tree with 1000 bootstrap replicates based on 49 common protein-coding genes from 29 complete chloroplast genomes of fabales. *Oxalis corymbosa*, *averrhoa carambola*, and *O. drummondii* are selected as an outgroup. Bootstrap values are shown at the nodes. Bootstrap supports were calculated from 1000 replicates. The red marker identifies the species investigated in this study. The following complete chloroplast genome sequences and accession IDs were used: *Trifolium alexandrinum*, MN857160 (Xiong et al. 2020); *glycyrrhiza glabra*, KF201590 (Kang et al. 2018); *wisteria sinensis*, KT200359; *abrus pulchellus*, MT328396 (Xu et al. 2022); *Vigna unguiculata*, NC_018051; *ammopiptanthus nanus*, KY034454; *lupinus westianus*, NC_036487; *arachis duranensis*, MK144822; *A. villosa*, MK144825; *A. helodes*, MK144826 (Wang et al. 2019); *acacia scalena*, LN885311; *A. gibbosa*, LN885271; *A. jibberdingensis*, LN885279 (williams et al. 2016); *adenanthera microsperma*, NC_034986; *bauhinia blakeana*, MN413506; *cadellia pentastylis*, NC_047334; *suriana maritima*, NC_047313; *quillaja saponaria*, NC_047356; *polygala hongkongensis*, MZ707521; *P. japonica*, MT762167 (Zou et al. 2021); *P. tenuifolia*, MT221251 (Lee et al. 2020); *P. arillata*, MK552120; *P. fallax*, MT762166; *lathyrus japonicus*, KJ806195; *medicago hybrida*, NC_027153; *M. papillosa*, KJ850241 (Yan et al. 2017); *astragalus mongholicus*, KR296789 (choi et al. 2016); *caragana kozlowii,* KX349219; *dalbergia cultrata*, NC_044117 (Liu et al. 2019); *oxalis corymbosa*, NC_048890 (Zhou et al. 2021); *O. drummondii*, NC_043802; *averrhoa carambola*, NC_033350.

DNASP and mVISTA were utilized in order to identify sequence variations and to compare the differences present within *Polygala* chloroplast genomes. Our analysis showed the pi value of nucleotide diversity fell within the range of 0 to 0.22041 (Figure S4). Further analysis revealed that seven regions, namely *atpH*-*atpI*, *rps4*-*accD*, *ycf15*-*ycf1*, *ndhF*-*rpl32*, *rpl32*-*ccsA*, *ndhE*-*ndhG*-*ndhI*, and *ycf1*-*trnN*, exhibited relatively high variability and were recognized as potential molecular markers or species-specific barcodes for phylogeny and species identification within the *Polygala* genus. Notably, the hyper-variable regions were primarily located in the LSC and SSC regions, while the intergenic regions showed a higher level of variability than that of the internal region of the genes. Besides, mVISTA was similarly used to conduct visual analyses of the five chloroplast genome sequences, and the results obtained were in agreement with those achieved through DNADP (Figure S5).

To explore the phylogenetic relationship in *P. fallax*, 29 species from Fabales and 3 from Oxalidales (*Oxalis corymbosa*, *Averrhoa carambola*, and *Oxalis drummondii*) as an outgroup were used to construct an ML phylogenetic tree. The phylogenetic analysis showed that *P. fallax* clustered with other *Polygala* species under a strong bootstrap. *P. fallax* was determined to be closely related to *P. arillata* (Figure 3). Our results support the conclusion that both *P. fallax* and *P. arillata* belong to Subgen. *Chamaebuxus*.

## Discussion and conclusion

In this study, the chloroplast genome of *P. fallax* (MT762166, 164,687 bp) was sequenced, assembled, and annotated. Our results are highly consistent with the previously reported *P. fallax* plastome (MN243712, 164,697 bp; Zhang et al. 2020), exhibiting high synteny and >99% sequence identity. Using DnaSP v6, we identified 42 SNPs and 8 indels, primarily located in the non-coding regions of the LSC region, reflecting limited intraspecific variation likely driven by geographic differentiation.

Notably, the SSC region in our assembly is significantly contracted (8,283 bp) compared to typical angiosperms, while the IR regions have expanded to 36,526 bp. We re-verified this atypical quadripartite structure through high-depth read mapping and boundary analysis using CPGView. This structure closely aligns with other *Polygala* chloroplast genomes (Zhang et al. 2020; Ma et al. 2021; Zou et al. 2021), and is consistent with the features observed in the previously reported *P. fallax* (MN243712) and its close relative *P. arillata*. This conservation suggests that such structural modifications represent a stable genomic signature within certain *Polygala* lineages rather than an assembly artifact. Consequently, these stable structural variations in the plastome provide valuable markers for further phylogenomic and evolutionary studies within the Polygalaceae family.

*Polygala* is a large, heterogeneous genus with species ranging from herbaceous to shrubby forms, and its classification remains contentious. It is broadly divided into the New World clade (NWC; 213 species in three sections) and the Old World clade (OWC; ∼349 species in 11 sections) (Abbott 2009). Morphologically, *P. fallax* and *P. arillata* are classified in Subgenus *Chamaebuxus* as woody shrubs, while *P. hongkongensis*, *P. japonica*, and *P. tenuifolia* belong to herbaceous Subgenus *Polygala*—a division largely supported by molecular data (Eriksen 1993; Abbott 2011). Comparative analysis of five *Polygala* chloroplast genomes identified seven highly variable regions (*atpH*-*atpI*, *rps4*-*accD*, *ycf15*-*ycf1*, *ndh1*-*ndh2*, *rpl32*-*ccsA*, *ndhE*-*ndhG*-*ndhI*, and *ycf1*-*trnN*), with nucleotide diversity (π) up to 0.22041. These hypervariable loci, mostly intergenic spacers in the LSC and SSC regions, serve as promising molecular markers for species discrimination—similar to barcoding approaches in *Salvia* (Cui et al. 2020)—and offer valuable tools to resolve taxonomic uncertainties exacerbated by morphological plasticity and convergent evolution in *Polygala*.

Our phylogenetic analysis of 49 shared protein-coding genes from 29 Fabales species strongly supports the current taxonomy of *Polygala: P. fallax* clusters with *P. arillata*, while the herbaceous species (*P. hongkongensis*, *P. japonica*, and *P. tenuifolia)* form a separate, well-supported clade. This clear split between Subgenera *Chamaebuxus* and *Polygala* corroborates earlier studies based on plastid (*trnL-F*, *rbcL*, *trnK-matK*) and nuclear *ITS* data (Abbott 2011; Pastore et al. 2019). The concordance between our chloroplast genome-based phylogeny and prior multi-locus analyses highlights the value of complete chloroplast genomes in resolving deep evolutionary relationships in taxonomically complex groups. These findings clarify the phylogenetic position of *P. fallax* and provide a foundation for developing molecular markers in *Polygala*.

## Supplementary Material

S4.tif

s1.tif

S2.tif

S3.tif

S5.tif

## Data Availability

The genome sequence data that support the findings of this study are openly available in GenBank of NCBI at (https://www.ncbi.nlm.nih.gov/) under the accession no. MT762166. The associated BioProject, SRA, and Bio-Sample numbers are PRJNA681297, SRR13161063, and SAMN16948892 respectively.
