# Supplementary figures and images for: The complete chloroplast genome of Polygala fallax hemsl. (polygalaceae), a medicinal plant in China
Source: Mitochondrial DNA B Resour. 2026 Jun 1;11(7):791–6. doi: 10.1080/23802359.2026.2677954 (PMC13228175; doi:10.1080/23802359.2026.2677954)

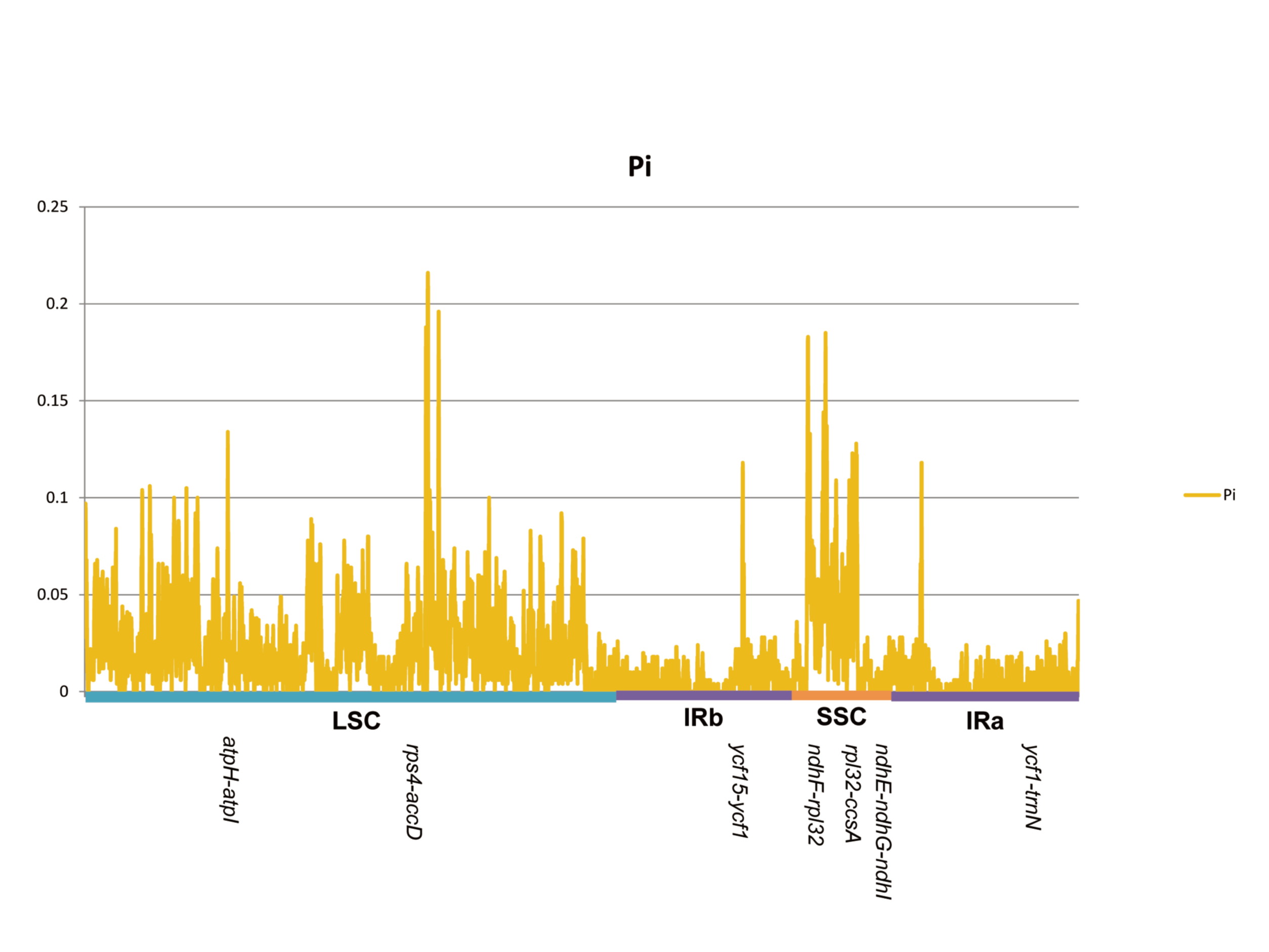

Supplement: S4.tif [file TMDN_A_2677954_SM1406.tif]

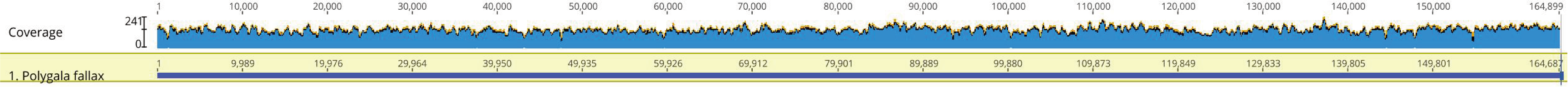

Supplement: s1.tif [file TMDN_A_2677954_SM1405.tif]

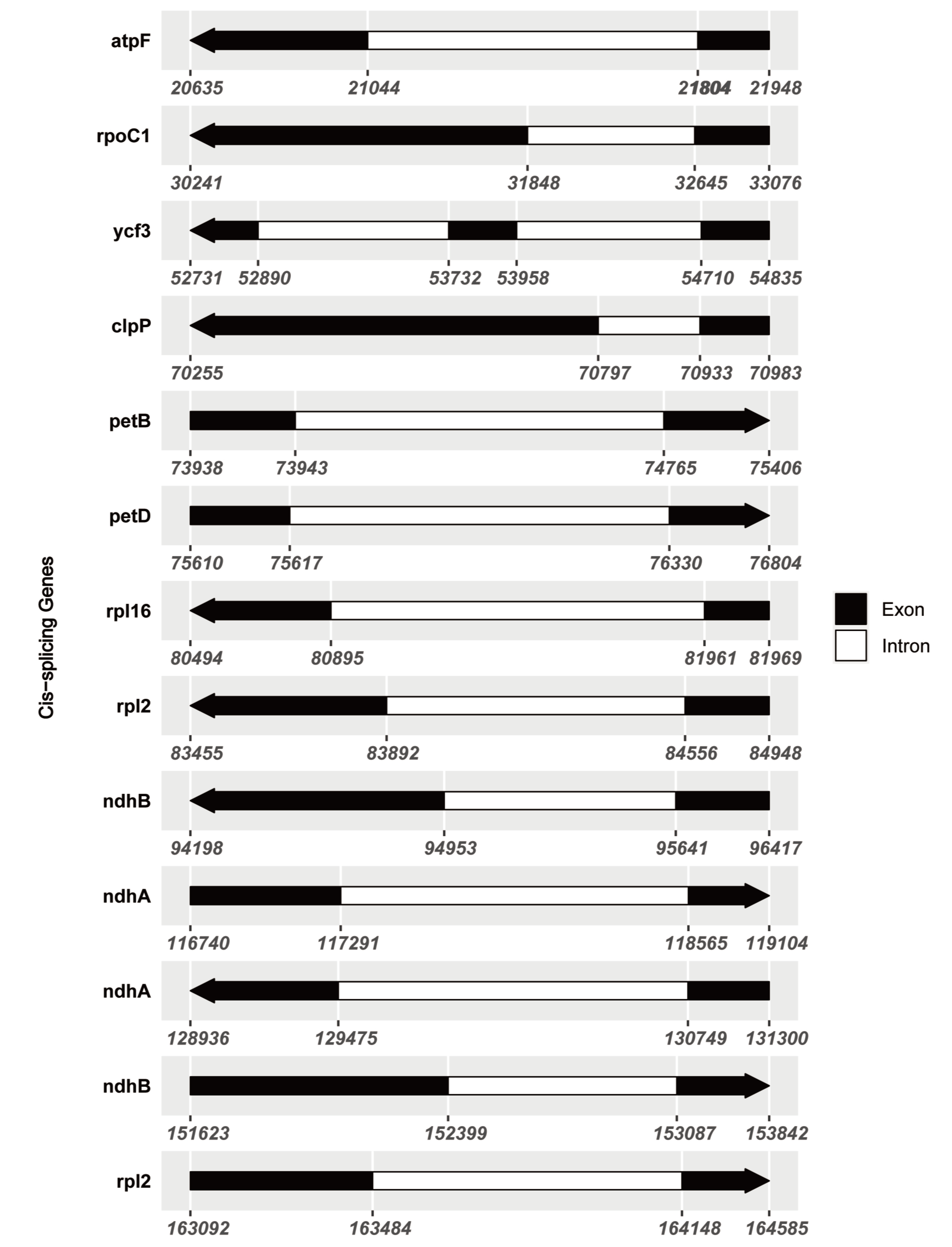

Supplement: S2.tif [file TMDN_A_2677954_SM1404.tif]

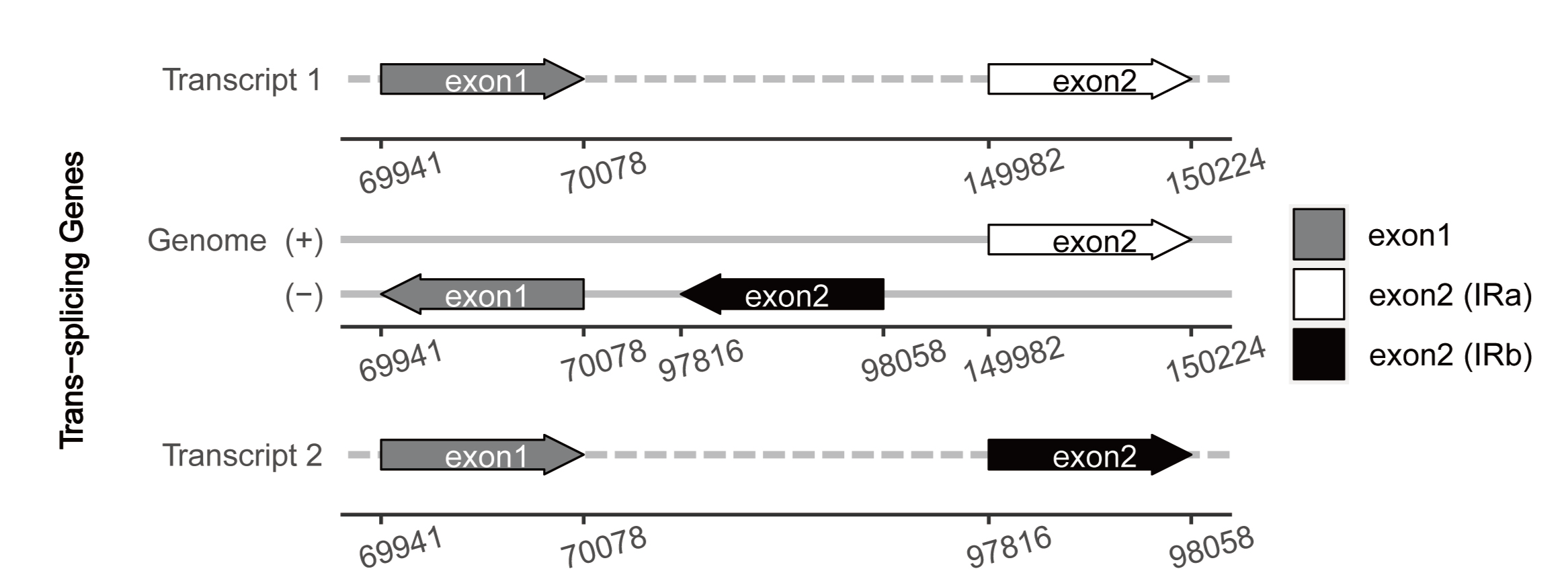

Supplement: S3.tif [file TMDN_A_2677954_SM1403.tif]

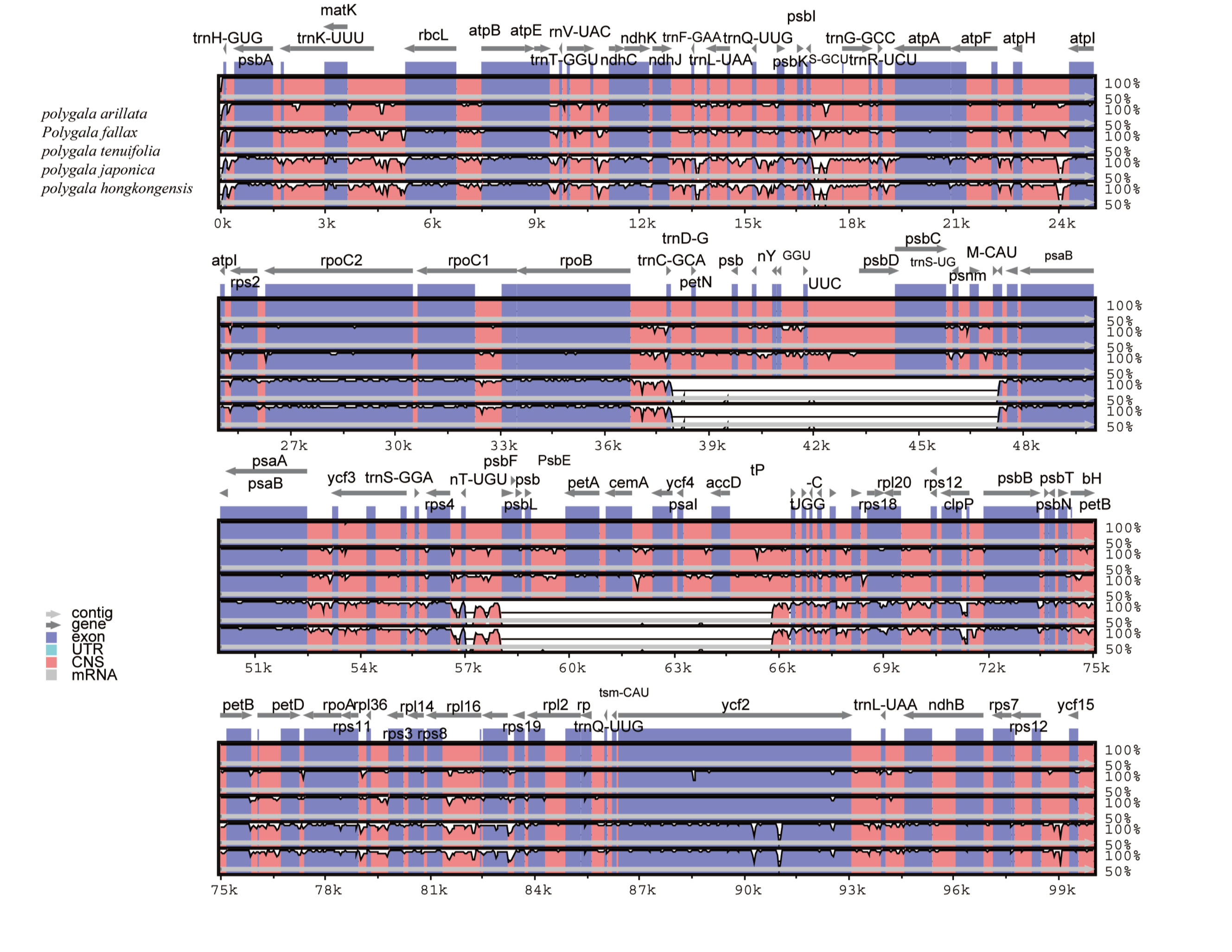

Supplement: S5.tif [file TMDN_A_2677954_SM1402.tif]
